# Pharmacists’ Perceptions on Nutritional Counseling of Oral Nutritional Supplements in the Community Pharmacy: An Exploratory Qualitative Study

**DOI:** 10.3390/pharmacy11020078

**Published:** 2023-04-20

**Authors:** João Gregório, Patricia Tavares, Emilia Alves

**Affiliations:** CBIOS—Universidade Lusófona’s Research Center for Biosciences & Health Technologies, 1749-024 Lisbon, Portugal

**Keywords:** malnutrition, pharmacy services, nutrition consultations, multidisciplinary collaboration, patient follow-up, thematic analysis

## Abstract

Malnutrition has important health impacts, especially in the elderly. Oral nutritional supplements (ONS) are effective strategies to help balance the nutritional needs of malnourished persons. Multiple ONS are available at community pharmacies, enabling pharmacists to have the possibility to implement strategies for prevention and monitoring of malnourished patients. The aim of this study was to characterize the experience of community pharmacists with the counseling and follow-up of users of ONS. A sample of 19 pharmacists from 19 different community pharmacies were interviewed. Apart from dispensing ONS to support patients that are preparing for diagnostic tests, the most frequently mentioned clinical condition for ONS counseling was malnutrition and dysphagia. When pharmacists consider dispensing ONS, three themes emerge: patient care, related to counselling tailored ONS to each patient’s needs; interprofessional collaboration, with a special focus in the collaboration with registered dietitians; and training and education on ONS, looking to improve their knowledge and skills in ONS counselling and follow-up. Future studies exploring new forms of interaction between pharmacists and dietitians in this context should be developed, aiming to determine the workflow of an interdisciplinary service addressing the needs of community dwelling malnourished patients.

## 1. Introduction

Malnutrition, a common process in the elderly and in cancer patients, which results from a lack of nutrient intake or absorption, leads to changes in body composition and impaired clinical outcomes [1,2,3]. One of the effective strategies to help balance the nutritional needs of malnourished persons is the use of oral nutritional supplements (ONS), as a nutritional intervention [4,5,6].

ONS are homogeneous mixtures of nutrients balanced for higher caloric intake, classified as food for special medical purposes to control disease-related malnutrition, that are commercially available for oral administration [7]. Administration of ONS has been shown to improve nutritional intake and nutritional status in individuals who are undernourished or at risk of undernutrition and to reduce clinical complications [3,8,9]. ONS are often nutritionally complete, meaning that when consumed in adequate quantities they can provide all essential nutrients (macronutrients along with essential micronutrients) to be a sole source of nutrition, which may not be achievable through a regular diet [3].

Nutritional interventions, consisting of dietary advice alone or in combination with an ONS, are considered an important cornerstone in the treatment of disease-related malnutrition [6]. ONS prescription includes the following criteria: (1) nutritional risk screening or nutritional assessment; (2) identification of the possible etiology of malnutrition; (3) description of the objectives of the ONS prescription and establishment of the desired outcome [10]. Nevertheless, the prevalence of inappropriate prescription of ONS is relevant [11]. The prescription of ONS is not always based on medical and/or nutritional advice. Inappropriate prescription of ONS exists when there is no clear clinical indication (overprescription), in cases of overdose or prolonged duration without clinical foundation (incorrect prescription) and in case of absence of ONS therapy with a clear clinical indication (underprescription) [12]. Moreover, due to the availability of such products in the community pharmacy market, the counselling and dispensing of ONS is often the responsibility of a community pharmacist, most of the time without any prescription whatsoever. In addition, patient compliance is generally low for ONS intake, thus potentially affecting their nutritional status, which suggests that patients receiving ONS should be targeted for both adequate counselling and additional adherence monitoring [13].

Portugal is currently one of the few countries where registered dietitians can carry out nutrition consultations in community pharmacies [14]. The introduction of nutrition consultations by registered dietitians in the community pharmacies has added value for all community care services, due to better access and the ability to contribute to health and well-being promotion [15,16]. These consultations have the potential to contribute to the implementation of health interventions with proven effectiveness, namely in the areas of maternal health, weight control, cholesterol, glycemia and blood pressure control. Community pharmacists have the possibility to implement strategies for prevention, health promotion and patient monitoring, namely in the area of malnutrition [17,18]. Through counseling, pharmacists can raise awareness for the importance of using ONS in tackling malnutrition and provide all the necessary information about its proper use, specifically side effects, posology, safety, interactions, precautions, preservation and storage conditions [19]. Nevertheless, no data are yet known about the impact of the community pharmacist in the follow-up and monitoring of nutritional interventions aimed at malnutrition, which could be a topic of interest to investigate. In order to contribute to this topic, the present study was designed to characterize the experience of community pharmacists with the counseling and monitoring of patients or users of ONS. To achieve this aim, three specific objectives were outlined, namely, to understand pharmacists’ knowledge of ONS; to describe the experience of counseling and follow-up of ONS users; and to identify what sources of information pharmacists use to know about ONS.

## 2. Materials and Methods

Given the novelty of the topic in this specific practice setting, an exploratory qualitative approach is the preferred methodology. In fact, there is little research on the use of ONS in community pharmacy, although some evidence already exists on the role of primary health care in the management of malnutrition in the community [12,20].

To implement this qualitative approach, a desk review on the topic was previously carried out aiming to screen the literature to understand the current state of the art and to understand which dimensions of pharmacists’ activity would be under investigation. PUBMED was searched, using Boolean operators to combine terms, such as “malnutrition”, “interventions”, “primary care”, “community pharmacists”, “dietitians” and “oral nutritional supplements”. This initial review informed the design of an initial interview script, consisting of 27 questions, which was pilot tested with three community pharmacists. After this pilot, three questions were changed and four questions withdrawn, resulting in the final version to be used (Appendix A). 

The population of interest were community pharmacists of the district of Lisbon, Portugal. The sampling was conducted according to the snowball sampling method, i.e., the selection of the subjects that make up the sample is made through nominations suggested by the respondents [21]. As inclusion criteria for this study, the contacted pharmacists had to work in community pharmacy, in a rural or urban setting, with a minimum of three years of continued and recent professional experience in community pharmacy. The minimum of three years of experience was considered important since it allows time for the professional to have multiple experiences on the subject from which they can report. Pharmacists who did not meet these inclusion criteria would not be invited. 

The interviews took place during May and June 2021, over Zoom© videoconferencing platform (version 5.6.6, Zoom Video Communications, Inc., San Jose, CA, USA) [22]. Zoom© is useful since it is simple to use and includes recording features to help with interview transcribing [23]. Two initial community pharmacists that fulfilled the inclusion criteria, acquainted to the researchers, were contacted and accepted to participate. All interviews started with the explanation of the study’s purpose and interviewees were given an informed consent statement to allow for the audio recording, ensuring anonymity and destruction of the audio after transcription. After the request for informed consent was accepted, the interview proceeded. At the end of the interview, the researcher asked for the contact details of two other professionals who could potentially participate, who were then contacted and, if they expressed willingness to participate and fulfilled the inclusion criteria, the interview was scheduled for a more convenient date and time. This procedure continued throughout the data collection period. This sampling technique allowed 19 interviews with pharmacists from 19 different pharmacies.

The audio recordings of the interviews were transcribed verbatim by the interviewer (PT) immediately after the interview, limiting memory bias. Preliminary thematic analysis of the transcriptions was performed by one researcher (PT), organizing the transcriptions and initial themes into an MS Excel^®^ spreadsheet. Afterwards, another researcher (JG) independently reviewed all the transcripts and produced more codes and themes. Finally, the themes were reviewed and discussed by all authors, until a set of final themes was identified. Descriptive statistics of the quantitative data collected (i.e., age, years of experience, frequency of themes) were also performed. The study protocol was submitted and approved by the Institutional Ethics Committee of Universidade Lusófona de Lisboa (CE. ECTS/P06-22).

## 3. Results

Nineteen pharmacists were interviewed, eighteen female and one male. Participants’ ages ranged from 25 to 43 years old, with an average of 32 years old (Table 1). The mean years of experience of these professionals in community pharmacies was 7 years. Regarding the years of permanence in the same pharmacy, the range was from 1 to 19 years with a mean of 7 years. Twelve (63%) respondents indicated working in a pharmacy in an urban setting while the remaining (n = 7) declared working in a rural setting.

Three quarters of the pharmacists said that the pharmacy where they work has nutrition consultations, all of them performed by registered dietitians. In these pharmacies, 38% of the consultations were exclusively focused on weight loss, while the remaining addressed other topics, such as clinical nutrition, body mass increase, dietary guidance for vegetarians and breastfeeding. Regarding the frequency of ONS counseling, it ranged from daily to monthly, with 42% of pharmacists stating that they provide counseling on ONS at least once a week. 

All respondents said that their pharmacy had ONS for sale, even in those pharmacies who did not have a nutrition consultation. Pharmacists stated a wide variety of reasons to counsel or sell these ONS (Table 2).

As expected, the main reasons to counsel ONS were directly related to their clinical indications. As for the information provided in this counseling, all pharmacists declared advising on the daily intake and time of administration. Only half informed about the storage precautions and need of refrigeration, and only two would give information on the duration of the therapy.

When counseling an ONS, pharmacists worried about specific pathologies or clinical situations that may impact their counselling protocol. The most frequently mentioned clinical condition to be considered during the ONS counseling was diabetes (84%), followed by renal insufficiency (47%) and oncological/cancer problems (37%). Dysphagia (26%) and swallowing problems, malnutrition, and bedridden patients (16%) were also mentioned. Other pathologies that deserve pharmacists’ attention when counselling ONS were liver failure, pressure ulcers or bedsores, patients who are tube fed, patients with neurological diseases (Alzheimer’s and Parkinson’s) and polymedicated patients. 

From our data analysis, three main themes related to the role of pharmacists in counselling ONS emerged: how and why to counsel and follow-up ONS consumers; ONS consumers follow-up as an interprofessional collaboration; and pharmacists’ training and education needs about ONS. In the following sections details are provided to each of these themes.

### 3.1. Patient Care: ONS Counselling and Monitoring

After counselling an ONS, all respondents considered it important to monitor patients on ONS, mainly to assess its evolution but also to increase personal trust in the product they were counseling or to increase customer loyalty to the pharmacy. The feedback given by the patient/client after the sale allows an increase in confidence in the product and in the advice given.

“Yes, I think it’s important, even to know if it’s working, if the patient is improving. In this way you also gain more confidence for new counseling. It is always an added value for us pharmacists to know if it is working or not, if it works it means that the supplement is effective and that the person is improving, which allows us to also have more confidence in the product and advise it more often.”

3ED, F, age 33.

Confidence in the product and in their own professional abilities seems to play an important role when counselling an ONS. This is most evident with cancer patients, where pharmacists considered that it is safer for this type of patient to have a medical prescription before coming to the pharmacy, thus also facilitating the counseling and follow-up of the patient. They were unanimous in recommending that this type of ONS be taken as directed by the product itself:

“Oncology patients and dietary supplements…. I don’t usually counsel. I always ask to talk to the doctor first, because with cancer patients, I don’t know what kind of treatments they are doing and although there are these kinds of supplements not all supplements are suitable for oncology. (…) I’m always very afraid.”

11JO, F, age 27.

As for how to perform patient monitoring, most pharmacists said they assess the patients when they return to the pharmacy. Only two pharmacists said they assess proactively (i.e., specifically scheduling an appointment with the pharmacist after a certain period). Two pharmacists indicated that they did not consider follow-up necessary, or if it is necessary, the registered dietitians should provide it:

“I think it’s important, and we follow up on this by waiting for the patient to return to the pharmacy (…) and we then ask “so how are you doing?”, as a way of understanding if everything is going well or not.”

2MÇ, F, age 30.

“(…) we know people relatively well, and when we leave the pharmacy and walk around in the street, we also meet them and we also ask how they are, if there are results, if they are feeling well (…).”

9AA, F, age 28.

Most of the pharmacists interviewed considered themselves to be the main facilitator for patient follow-up, possibly recording follow-up information in a dedicated information system. Another important facilitator for patient follow-up was the existence of a specific consultation for these patients. Lack of time was referred to as one of the barriers, while two pharmacists considered that, since the pharmacy is located in a small neighborhood, where the relationship with the patients is closer, the registration of information was not needed:

“It could be important to have the information registered, but it would be more complicated, it would already be too much, in my opinion. But the time factor is important. I would love to have time to keep up with everyone, but this in an ideal point of view and it’s not realistic.”

5RI, F, age 27.

The majority of the respondents (53%) pointed to the high cost of ONS as the main barrier to adherence, which in turn contributes to lower counseling and sales opportunities. Also hindering counseling is the lack of knowledge regarding several ONS characteristics, such as dosage, duration of therapy and possible adverse effects in special cases, such as children, pregnant women, polymedicated patients and intolerances. The range of offers also makes it difficult to choose the ONS most adapted to the individual patient’s needs:

“The price of this type of supplement is very high, which sometimes makes adherence to this type of therapy more difficult, especially for the elderly, who usually spend a lot of money on medications. Taking into account that these supplements can be an important help in the improvement and well-being of these people there should be an aid to support these costs, or even be reimbursed, given its relevance in the health of the user.”

3ED, F, age 33.

“We can sometimes advise to replace a meal, but people end up not adhering to it because the costs are so high.”

8AG, F, 25 years old.

### 3.2. Interprofessional Collaborations

Two other health professionals were referred to as having an important role in ONS prescription, counselling, and follow-up: physicians (being either general practitioners (GP) or specialists) and registered dietitians. Physicians were usually evoked to deal with difficult cases, such as cancer patients, while dietitians were mostly recognized as a useful ally in both patient counselling and follow-up, using the experience of nutrition consultations as a benchmark:

“In that case [cancer patients], we always try to have that kind of counseling done more by the doctor than by us, for safety’s sake, because they are more fragile patients with certain specific needs (…). We advise them, we let them know what is available and what the benefits are, but we say that the best thing is to talk to the doctor (…) so that we don’t take risks with this type of patient, because they are a type of patient that needs other care. If it comes with a doctor’s indication we advise the basic care to have.”

8AG, F, age 25.

“Just as there is the nutrition consultation, there could be a follow-up consultation with the patient, according to his/her needs, making a record of his/her condition, if he/she is better or worse.”

3ED, F, age 33.

### 3.3. Training and Education Needs 

Only two pharmacists reported feeling confident enough to counsel ONS without further training. Three pharmacists reported not having had any training on ONS. Of the pharmacists who received training on ONS, the majority did not feel that they had acquired all the necessary training on this type of supplement. The reported reasons for this need for training seem to be based on the increased prevalence of these products in the pharmacies’ product portfolio. In general, training was provided in the pharmacy itself, through representatives of the ONS manufacturers:

“I really think that there should be more training on this subject, because, in fact, it’s something that at least in pharmacy, has been increasing the number of sales, especially because we have an older population, many more pathologies (…) the basis for better counseling is really training, I think that should be part of it. To always keep up to date with the latest news about interactions and adverse reactions, (…) and it’s something that I don’t happen to know, and I always think it’s important.”

10PA, F, age 26.

One of the strategies adopted to overcome the lack of training was asking more experienced colleagues. In addition, the scarcity of information available in the pharmacies’ information systems was also mentioned, reflecting their inadequacy for this purpose:

“Essentially, the lab’s website or product website to get the most information or else working with SIFARMA^®^ [pharmacy information system], although I don’t think supplements have much information in the computer system. Because I don’t have any other sources of information, we go around making questions to each other.”

10PA, F, age 26.

“SIFARMA^®^, but it has nothing good, lab’s websites and older colleagues who know!”

11JO, F, 27 years old.

“The information available is scarce. I want to believe that the information, especially from the laboratories’ webpage, is information you can trust. SIFARMA^®^ helps a little bit but it is very limited, you can’t have any level of specification, because sometimes you can even have a “slight interaction alert” with something, but is that slight interaction significant or not?!”

1DG, F, 31 years old.

## 4. Discussion

In the thematic analysis here presented, three main themes were found. The first theme concerns the technical component of counseling, i.e., knowing how to advise the right product to a specific patient. This theme includes other sub-themes, such as the confidence in the counseling, which increases with the frequency of ONS dispensing; patient compliance to the ONS, reflected in the perception that the price of these ONS may hamper patient compliance, leading to lower efforts in provision of a structured follow-up; and the differing approaches to follow-up where there seems to be a dichotomy between the “should do”—as a professional’s duty—and the “can do”—identifying lack of time and inadequate information systems as barriers to this duty. The second theme was about the interprofessional collaborations pharmacists perceive as valuable for these patients. Here, feelings of insecurity when counseling certain patients drives pharmacists to refer patients to physicians (GP or others) in more difficult cases. Moreover, a special importance was attributed to the dietitian due to their knowledge and skills, which drove pharmacists to consider that dietitians’ presence or collaboration would facilitate treatment strategies and its outcomes. Finally, the last theme was the perceived need for more training and education about ONS, either at undergraduate level or postgraduate level, but more often in a professional setting, aiming to improve their knowledge on the subject but also on how to provide better follow-up.

Regarding counseling, pharmacists generally understood its importance, but only a minority proactively asked patients to return to the pharmacy for follow-up. When pharmacists considered the monitoring and follow-up of a patient on ONS, they did so considering three dimensions: (1) a patient care dimension, mainly focused on identifying how a patient evolves with a given intervention, highlighting the caring role of the pharmacist; (2) a professional development dimension, concerned with improving their knowledge through experience with the use of a product or intervention (akin to the clinical reasoning cycle [24]—they know what works, and for whom, and they adjust their future advice with this information, which goes beyond the information that is provided by the ONS’s companies); (3) a commercial dimension, related to the perceived sustainability of the pharmacy’s business. They were concerned with the customers/patients returning to their pharmacy, to purchase a product, instead of choosing another pharmacy as an alternative. These three dimensions reflect factors affecting community pharmacists’ provision of pharmacy services [25], and are consistent with evidence showing that patient follow-up in community pharmacies does not occur in the desired proportion [26], which conflicts with the general consensus that this function is vital for the profession and the daily practice of pharmacists [26]. The need to record information to support follow-up was identified, but lack of time was cited as the main reason for not doing so, alongside feeling that structured follow-up was not necessary because of the pharmacy’s insertion in a small community/neighborhood. Lack of time is many times cited as a barrier to the provision of any structured pharmacy service, such as patient follow-up [26,27], rooted in high workloads and inadequate pharmacy workflow [28,29], and not surprisingly it is also mentioned as an impediment for nutrition care services [17]. 

In this group of pharmacists, the concern to detect situations concomitant with malnutrition, namely associated with diseases such as diabetes, renal failure, or dysphagia, was notorious. In the case of cancer patients seeking ONS, pharmacists’ choice was to immediately refer them to a physician, conveying a feeling of insecurity in counseling this type of patient. This insecurity and lack of confidence in the indication of an ONS can be partly justified both by patients’ perceptions that the pharmacist’s role should be limited only to guidance regarding the use of the medication, and the lack of training and education on nutritional care subjects [17,18], reinforcing their aversion to risk and the need for validation [30,31]. 

Alongside physicians, pharmacists also mentioned registered dietitians as an alternative, as others have also done elsewhere [20,32]. However, there seems to exist some confusion about the role general practitioners and dietitians play when addressing patients with malnutrition. The correct prescription of the ONS must be preceded by a nutritional assessment that is the responsibility of a registered dietitian in an autonomous process [33]. Arends and colleagues [34] mention the special importance of registered dietitians in monitoring patients since they have the knowledge to outline the appropriate strategies throughout the treatment. It should be noted that, in Portugal, ONS counseling is a registered dietitian’s specific clinical skill, which is not always recognized by other health professionals [35]. Moreover, registered dietitians also have better explaining abilities about ONS than pharmacists [32]. In the case of Portuguese community pharmacies, most pharmacies have installed capacity to advance a new nutritional intervention service for patients on ONS, since most pharmacies have a collaboration with a registered dietitian. In these consultations, mainly focused on weight loss programs, the participation of the community pharmacist is limited to the moment of sale/dispensing of the ONS brand that many times supports the consultations’ costs [15,16]. The increasing prevalence of these consultations has contributed to highlighting the pharmacist–dietitian collaboration as one of the best examples of interprofessional and multidisciplinary collaboration in a community or primary care setting. Thus, the construction of a synergistic pharmacist–dietitian relationship can represent a turning-point for health promotion at community pharmacy level. As a matter of fact, this relationship has already been pinpointed in other studies as crucial for the optimal use of ONS or other nutritional and dietary management [17,20,32]. As such, the most viable scenario for a future service workflow should start with an initial evaluation and prescription of the ONS by the dietitian, followed by the dispensing of the ONS to the patient by the pharmacist. Follow-up could be ensured either by the dietitian or the pharmacist, depending on what parameters should be monitored (e.g., weight and body mass index can be measured by any person, while brachial or calf circumference and measures of skinfold thicknesses are more specific and should be obtained by trained professionals, in this case dietitians). Evidence suggests that proactive attitudes by pharmacists in determining parameters, such as weight, muscle mass index and fat mass index, would provide a better health service to the patient, which would potentially benefit a tailored nutritional counseling [1,19]. Nevertheless, the need to increase knowledge about the functions and competences of the health professionals involved in caring for patients needing nutritional interventions remains.

### Limitations and Future Directions

Although a small sample size, which can be considered one of the limitations of this study, the interviewed sample of professionals in this study reflects the demographics of the profession, i.e., a young, mostly female population, concentrated in urban areas [36], even though one third of the interviewees were from community pharmacies in rural areas. In this sample, only one male pharmacist was interviewed, which can be considered a high risk of bias. Nevertheless, since the sample is not representative of the profession, the results presented here can only be seen as an exploratory analysis that may pave the way for further research. Moreover, by not employing a predefined framework for thematic analysis in this exploratory research, the validity and reliability of the analytical process may have been hampered and it is possible that significant themes or patterns in the data may have gone unnoticed. However, we feel confident that the opinions and perceptions expressed by this group of pharmacists are the mainstream view of most pharmacists in Portugal. 

It would be of special interest to develop new studies exploring new forms of interaction between pharmacists and dietitians, as well as studies that evaluate and determine which tools are most important for the development of information systems that allow for the follow-up and monitoring of patients using ONS therapy in a collaborative approach to nutrition care services in the community.

## 5. Conclusions

The results of this study allow us to conclude that it is easier for pharmacists to counsel and dispense ONS than to continue the follow-up after that counselling. To implement a successful follow-up, pharmacists suggest deepening an interprofessional collaboration with dietitians and refer the need for training and education on ONS subjects and follow-up methodologies and skills.

## Figures and Tables

**Table 1:** Characterization of the interviewed community pharmacists (n = 19).

Gender	Age (y.o.)	Years of Experience	Pharmacy Setting
Female	31	5	Urban
Female	30	8	Rural
Female	33	6	Urban
Female	33	11	Rural
Female	27	4	Urban
Female	25	3	Rural
Female	26	3	Rural
Female	25	3	Urban
Female	28	5	Urban
Female	26	3	Urban
Female	27	4	Urban
Female	30	5	Urban
Female	38	8	Urban
Male	41	12	Rural
Female	43	19	Rural
Female	31	8	Urban
Female	25	3	Urban
Female	42	15	Urban
Female	43	15	Rural

**Table 2:** Main reasons identified by the pharmacists as opportunities for ONS counselling and selling.

Counselling/Selling Reasons	Proportion of Pharmacists, % (n)
Diagnostic exams (colonoscopies, endoscopies)	37% (7)
Elderly, debilitated, bedridden persons	32% (6)
Malnutrition, low weight, weight loss	32% (6)
Dysphagia	32% (6)
Cancer patients	21% (4)
Expressed need of the person	21% (4)
Post-surgery/hospitalization	16% (3)
Loss of appetite	11% (2)
Diabetics	11% (2)
Pregnant women	5% (1)
Medical prescriptions	5% (1)

## Data Availability

The data presented in this study are available on request from the corresponding author, on reasonable request. The data are not publicly available due to privacy protection of the interviewees.

## Appendix A

### Interview Script

“This interview aims to assess the knowledge of pharmacists in counseling hyperproteic and hypercaloric oral nutritional supplements (ONS) in the community pharmacy. The questions asked refer only to hyperproteic and hypercaloric ONS indicated to meet the protein and energy nutritional needs in conjunction with the usual diet.

This study is part of the dissertation to obtain the Integrated Master’s Degree in Pharmaceutical Sciences of the Lusófona University of Lisbon. To better achieve the aim of this interview it is intended that you answer in an authentic way according to your acquired knowledge, in order to get to know better the reality of pharmacists on this subject. The audio of this interview will be recorded, all the data collected will be used solely for academic purposes, ensuring the anonymity of the interviewee and the pharmacy, and at the end of the study, all the audios will be destroyed.

Do you agree or disagree with the terms of this interview?

Thank you for your cooperation.

Date/time/code:

1. Gender:
2. Age:
3. Years of experience in community pharmacy?
4. Years working in the same community pharmacy?
5. In which area is the pharmacy located: rural or urban?
6. Does the pharmacy have nutrition consultation? Yes/No
7. Who performs the nutrition consultation?
8. In the nutrition consultation, do you know/discuss other topics that are not related to the weight loss diet? If yes, which?
9. Does the pharmacy have hyper-proteiic and hyper-caloric nutritional supplements for sale?
10. How often do you recommend one of these food supplements? Daily/2x a week/Every 15 days/weekly/monthly
11. What motivates you to recommend a dietary supplement to a patient? Why? How do you do it?
12. In your opinion, do you consider important to follow up the patient after the dispensing of these products? Why? How?
13. What conditions would facilitate the pharmacist’s role in this follow-up?
14. When you advise a dietary supplement, do you take into account the medical conditions of the patient? If yes, give examples
15. How would you advise one of these supplements to a cancer patient?
16. Do you have any training on hyperproteic and hypercaloric food supplements? If yes, where did you acquire it?
17. As a Pharmacist do you feel you have acquired the necessary training to counsel with confidence a food supplement?
18. How could you improve your knowledge for better advice on dietary supplements?
19. As a Pharmacist what difficulties or barriers do you feel when advising a dietary supplement? Why?
20. Do you feel adequately informed about interactions and side effects of food supplements?
21. In case of doubts in counseling, what sources (3) do you use?
22. In your opinion do you think that information on this subject is available and reliable?
23. Do you have any other suggestions or thoughts you would like to leave?”
